# Bias-Mitigated AI as a Foundation for Resilient and Effective Health Systems

**DOI:** 10.2196/88457

**Published:** 2026-02-23

**Authors:** Jarbas Barbosa da Silva Jr, Maureen Birminghamm, Ana Rivière Cinnamond, Eldonna Boisson, Mary Lou Valdez, Sebastian Garcia Saiso, Joao Paulo Souza, Myrna C Marti, Leah-Marie Richards, Javier Guzman, Jennifer Nelson, Karina Pesce, Ana Estela Haddad, James Fitzgerald, Ernesto Bascolo, Marcelo D'Agostino

**Affiliations:** 1 World Health Organization Regional Office for the Americas, 525 23rd St NW, Washington, DC, 20037, United States, 1 2029743301; 2 Inter-American Development Bank, Washington, DC, United States; 3 Hospital Italiano de Buenos Aires, Buenos Aires, Argentina; 4 Secretariat of Information and Digital Health, Ministry of Health (Brazil), Brasília, Federal District, Brazil

**Keywords:** primary health care, artificial intelligence, digital health, bias in artificial intelligence, AI

## Abstract

Artificial intelligence (AI) is rapidly reshaping the landscape of health care, from clinical diagnostics and disease surveillance to the prediction of individual health risks. Yet, its immense promise will only materialize if the tools we deploy work for everyone. Algorithms trained on incomplete or biased datasets risk embedding historical health disparities and can replicate patterns of uneven data representation, thereby limiting accuracy and generalizability across population groups. Addressing algorithmic bias should be treated as a core health quality standard, comparable in importance to safety and efficacy evaluations, to ensure consistent performance across all segments of the population. This paper aims to frame algorithmic bias in health-related AI as a quality, safety, and governance challenge for health systems rather than solely a technical problem for developers. It aims to inform policymakers, regulators, health system leaders, and developers by translating existing scientific evidence and regulatory guidance into operational governance considerations, with particular attention to the realities of low- and middle-income settings in the region of the Americas. This paper synthesizes existing knowledge and institutional experience into a practical, regionally grounded policy perspective. To operationalize this perspective, this paper first outlines the main forms of algorithmic bias relevant to health systems—including representation, measurement, aggregation, and deployment biases—and illustrates how each can emerge across the AI lifecycle. It then situates these technical challenges within the broader digital health context, where structural, commercial, and social dynamics may amplify inequities. This paper discusses the implications of biased data for emerging areas such as precision medicine before proposing a governance-oriented framework for bias mitigation that spans design, validation, deployment, and postmarket monitoring. It concludes with priority governance actions for policymakers, regulators, and health system leaders to embed fairness as a measurable component of health system performance.

## When Data Exclude, Health Follows: The Manifestations of Algorithmic Bias

The problem of bias is not one of malicious intent but of deep-seated scientific and structural oversight. While some of the examples discussed below originate in data collection practices or medical device calibration rather than in artificial intelligence (AI) models themselves, these upstream measurement biases are critical to understanding algorithmic bias in health. In practice, biased measurements and nonrepresentative clinical data are often used as training inputs for AI systems, allowing inequities embedded in devices or data standards to be inherited, amplified, and scaled through algorithmic decision-making. When data are nonrepresentative, bias often follows. Algorithms are only as sound as the data they are trained on, and when datasets fail to represent the full diversity of our populations across ancestry, gender, geography, and socioeconomic status, the resulting tools may perform well on average but systematically fail for underrepresented groups. This challenge has been documented in clinical settings and, in some cases, addressed, underscoring the need for improved model generalizability and data representation. The sources of bias are varied and can emerge at any stage of the AI lifecycle [1,2]. To strengthen the analytical structure, bias can be grouped into four main categories: representation bias, measurement bias, aggregation bias, and deployment bias. This taxonomy helps identify specific interventions at each stage.

A clear example is representation bias, where training data does not reflect the target population. An AI system designed to detect skin cancer, for instance, achieved high accuracy when trained predominantly on images of light-skinned individuals. However, its diagnostic accuracy was nearly halved when applied to lesions on Black patients, reducing diagnostic performance in populations where early detection is already more difficult due to historical underrepresentation in medical datasets [3]. The result is an algorithm that may systematically discount reported symptoms or predict poor adherence, automating and scaling existing disparities, including in areas such as pain management and diagnostic triage decisions [1,4]. These examples illustrate that even well-established clinical language can reflect historical assumptions, an important consideration for developers of data-driven tools. Similar lifecycle-based taxonomies have been proposed in the broader machine learning literature, which consistently identify data collection and representation as primary sources of algorithmic unfairness, alongside proxy feature selection, model training, and deployment-related feedback effects [5].

Measurement bias can be embedded in the very tools we use to collect data. The pulse oximeter, a ubiquitous medical device, has been shown to overestimate blood oxygen levels in individuals with darker skin tones, a flaw rooted in the device’s initial calibration on insufficiently diverse populations [1,6]. Although pulse oximetry bias is not, in itself, an AI-specific phenomenon, its relevance to this discussion lies in the fact that oxygen saturation data are frequently used in clinical datasets and predictive models, including AI-based risk stratification and decision support tools. When such measurements are systematically skewed for certain population groups, downstream algorithms trained on these data may reproduce or intensify these inaccuracies at scale. During the COVID-19 pandemic, this technical inaccuracy had profound implications, potentially delaying the recognition of severe hypoxemia and the delivery of critical care to already hard-hit communities [7]. Similar biases are found in clinical tools like spirometry equations that apply race-based “corrections” that may not be scientifically sound [6]. Similar considerations apply to race-based spirometry equations, where historically embedded assumptions in measurement standards can shape clinical datasets and, if left unexamined, be incorporated into AI-enabled models that rely on pulmonary function data for prediction or triage. In the Latin American and Caribbean region, where most medical devices are imported and not locally calibrated, this type of bias may be amplified, affecting vulnerable and remote populations that already face different barriers of access to specialized care.

Aggregation and deployment biases can emerge even with good data. Aggregation bias occurs when a single model is applied to a diverse population, ignoring crucial differences between subgroups. For years, cardiovascular risk models built predominantly on male data underdiagnosed heart attacks in women, whose symptoms can differ significantly [8]. Deployment bias arises when a tool developed in one context is applied to another without adaptation. Deployment bias can also appear in the prioritization of AI applications, where tools developed predominantly for specialized or hospital-based clinical contexts may inadvertently overshadow the needs of primary care and essential public health services, areas where data availability and research output are more limited. An algorithm trained in a high-resource tertiary hospital may perform poorly in a rural clinic with different patient demographics and resources [9]. This was also evident in the global response to COVID-19, where digital contact-tracing apps requiring modern smartphones and reliable internet inadvertently excluded older, poorer, and rural populations, the very groups most vulnerable to the pandemic’s impact [10,11].

It is important to note that many of the well-documented empirical examples of algorithmic and measurement bias cited in the literature originate from high-income settings, reflecting where AI evaluation and postmarket surveillance mechanisms are most established. This does not imply that such biases are less relevant in Latin America and the Caribbean. On the contrary, health systems in the region frequently rely on imported medical devices, digital tools, and AI-enabled applications developed and validated elsewhere, often without systematic local calibration or performance assessment across diverse populations. The relative scarcity of region-specific evidence therefore represents a structural evidence gap rather than an absence of risk, underscoring the need for stronger regional capacity in evaluation, governance, and postdeployment monitoring of AI-enabled health technologies.

## The Digital Context of Health

These technical biases do not exist in a vacuum. They are magnified by the emerging digital landscape, where structural forces, such as commercial and social ones, can shape and/or exacerbate existing disparities in health outcomes [12]. The same platforms that could deliver critical health information can also be vectors for dangerous misinformation or be used to market unhealthy products that may be particularly detrimental to vulnerable populations. Moreover, the vast datasets collected by technology companies, often without transparent consent, may be used in ways that deepen existing patterns of exclusion and social stratification. Addressing algorithmic bias in clinical tools is a critical first step, while also encouraging a digital ecosystem that balances innovation with transparent, responsible use of data in support of health objectives. Building institutional public-sector capacity for AI governance, including regulatory oversight, audit mechanisms, and digital literacy among health professionals, is essential to address health population needs, especially for low- and middle-income countries that face financial and human resource constraints.

## Precision Medicine Demands Precision in Data

These biases extend to the most advanced frontiers of medicine. Precision medicine, which aims to tailor care to an individual’s unique genetic, environmental, and lifestyle profile, is severely hampered by a profound lack of diversity in the foundational data. An estimated 80% of participants in genome-wide association studies—the research that underpins many genetic tests—have been of European descent, a group that comprises just 16% of the world’s population [13]. The consequences are alarming. The resulting genetic risk scores, used to predict the likelihood of developing diseases like cancer or heart disease, are most accurate for this single, well-studied group. For individuals of African, Asian, or Indigenous ancestry, these scores can be uninformative or, worse, misleading, limiting their clinical utility and the clinical relevance of advanced medical tools for diverse populations and reducing the full potential of precision medicine [12,14]. In Latin America, where more than half of the population identifies as Indigenous, Afro-descendant, or of mixed ancestry, underrepresentation in genomic research limits the accuracy and fairness of emerging AI-based diagnostic tools.

Expanding ancestral diversity in datasets is a critical prerequisite for making precision medicine more accurate, equitable, and clinically relevant across populations. Furthermore, investing in robust, representative data is a catalyst for discovery. Calls for increased diversity in precision medicine research often conflate multiple meanings of representation, spanning genetic ancestry, social identity, and inclusion objectives, which can lead to ambiguity in how datasets are constructed and interpreted [15]. Collecting pharmacogenomic data across diverse populations can prevent one-size-fits-all dosing errors and reveal novel population-specific genetic variants that could inspire new, targeted treatments [16,17]. For companies developing clinical AI tools and medical devices, ensuring reliable performance across all population groups reduces liability risks and facilitates regulatory approval, factors that support broader adoption and market sustainability. Establishing regional genomic research networks and public–private partnerships can help ensure that Latin America and the Caribbean are included in this new frontier of data-driven medicine.

## A Framework for Fairness: Bias Mitigation Across the Algorithm Lifecycle

Achieving bias-mitigated AI requires vigilance and intentionality at every stage. A proactive, sociotechnical approach is needed. First, the process must begin with inclusive design and data collection. This means moving beyond convenience sampling to deliberately curate datasets that represent the full spectrum of the target population. Health-related institutions and developers should partner with minority-serving clinics and community organizations to fill critical data gaps. Where data are scarce, techniques like synthetic data augmentation may be carefully explored as a temporary bridge, but they are no substitute for real-world representation [18]. In the region of the Americas, countries can leverage the Information Systems for Health Framework and the Pan American Highway for Digital Health Initiative to establish secure, interoperable, and bias-aware data ecosystems that enable such inclusivity.

Second, it is critical to implement rigorous bias checks and fairness metrics during model building and validation. It is not enough to measure overall accuracy. Performance must be audited by subgroup to identify any significant disparities in error rates. A model that is 95% accurate for one group but only 80% for another is not a sign of robustness; it highlights the need for improved performance across diverse population segments to ensure reliability and impact in health. These audits should be a standard requirement before any algorithm is deployed in a clinical setting [19].

Strengthening algorithm performance across population subgroups also supports domestic preparedness by cultivating a digitally competent, bias-aware health workforce. These efforts align with voluntary standards such as the AI Risk Management Framework developed by the National Institute of Standards and Technology and the Good Machine Learning Practice principles issued by the US Food and Drug Administration, ensuring that AI in health care is both innovative and reliable. Collaborative partnerships between industry, regulators, and health care providers are essential to ensure that these tools are not only clinically sound but widely accessible. Expanding population representation in health datasets is a prerequisite for scientific accuracy, improved generalizability, and reduced liability across AI-enabled medical systems. To operationalize fairness, governments could adopt measurable indicators such as subgroup parity rates, demographic calibration metrics, and public reporting of bias audit results as part of AI system certification or procurement. Fairness metrics are inherently context-dependent and may involve trade-offs between competing objectives such as sensitivity, specificity, and equity across population groups. No single metric or threshold is universally applicable across all clinical or public health use cases. As a result, the selection and interpretation of fairness measures should be informed by clinical context, population characteristics, and local health system priorities and should be complemented by human oversight and local validation.

Third, regulatory agencies should take the lead in advancing transparency and explainability in health AI systems. While voluntary frameworks, such as the Good Machine Learning Practices and the AI Risk Management Framework, are important early steps, they can lay the groundwork for future formal regulations with enforceable requirements and mandatory mechanisms. Developers should be required to provide clear documentation on data sources, demographic representation, and known limitations, often referred to as “datasheets for datasets.” This transparency will allow clinical institutions and oversight bodies to better evaluate algorithmic recommendations, ensuring these tools support, rather than replace, sound medical judgment. These actions align with global regulatory trends, including the EU AI Act, which mandates oversight for high-risk AI systems [20]. Regional adaptation of these principles through national AI oversight committees would promote harmonized, context-appropriate governance across the Americas.

Fourth, diverse multidisciplinary teams, including clinicians, data scientists, bioethicists, and representatives of affected communities, are essential to uncovering blind spots that homogeneous teams might miss. Engaging patients and community members can reveal contextual factors and potential harm that are not apparent from a purely technical perspective. Including gender experts, disability advocates, and Indigenous community representatives in these teams ensures an intersectional approach to fairness.

Finally, strengthening health authority stewardship with clear and strong governance capacities, supported by accountability mechanisms, is essential. Regulatory agencies should define enforceable requirements for algorithmic performance, transparency, and postmarket monitoring. In parallel, institutional review bodies, such as algorithm bias–resistant committees, can play a complementary role within health systems or clinical institutions by independently evaluating tools for bias, transparency, and contextual fit before implementation. Once deployed, ongoing monitoring through real-world bias audits is necessary to detect performance drift and emerging disparities over time. To maintain trust, there must also be well-defined pathways for redress when AI tools are found to cause harm. Governments can also establish national registries of AI systems in health, linking performance data, fairness audits, and postmarket surveillance to ensure accountability and transparency.

To move from principles to practice, governance-oriented actions are needed that can be realistically implemented across diverse health system contexts. Table 1 summarizes a set of priority governance actions that health authorities and regulators can use to operationalize bias mitigation across the AI lifecycle. These actions are intended to be a flexible policy toolbox that can be adapted to different levels of regulatory maturity and resource availability, with particular relevance for low- and middle-income settings. The table summarizes governance-oriented actions proposed in this policy perspective to support bias mitigation in AI-enabled health tools across design, validation, deployment, and postmarket monitoring stages. The actions are intended for health policymakers, regulators, and health system leaders, with particular relevance for low- and middle-income settings in the region of the Americas.

**Table 1.** Priority governance actions to mitigate algorithmic bias across the artificial intelligence (AI) lifecycle in health systems.

Governance lever	Priority action	Primary actors	Policy rationale
Transparency and disclosure	Require population-representativeness disclosures for AI-enabled health tools, including data sources, demographic coverage, and known performance limitations.	Regulators, ministries of health.	Enables informed decision-making, reduces hidden bias risks, and supports accountability in procurement and deployment.
Performance assessment	Mandate subgroup-level performance reporting for high-risk AI applications rather than reliance on aggregate accuracy metrics.	Regulators, health technology assessment bodies.	Identifies differential impacts across population groups and prevents masking of inequities.
Predeployment review	Establish multidisciplinary review mechanisms within health authorities to assess bias, transparency, and contextual fit prior to deployment.	Ministries of health, regulatory agencies, ethics committees.	Leverages existing institutional structures to identify risks before scale-up.
Local validation	Promote context-specific validation of AI tools, particularly when systems are imported or adapted from other countries.	Health institutions, regulators, implementers.	Reduces deployment bias and ensures relevance to local populations and care pathways.
Postmarket monitoring	Integrate bias considerations into postmarket surveillance using routine health data and real-world evidence.	Regulators, health system operators.	Detects performance drift and emerging disparities after implementation.
Data governance	Strengthen data governance and interoperability frameworks to improve data quality, disaggregation, and representativeness.	Governments, digital health authorities.	Creates the foundational conditions for equitable AI development over time.
Workforce capacity	Invest in digital literacy and AI governance competencies for regulators, clinicians, and public health professionals.	Ministries of health, academic institutions.	Enables informed oversight and responsible adoption of AI tools.
Procurement and financing	Link fairness, transparency, and accountability criteria to public procurement and funding decisions.	Governments, public purchasers, donors.	Institutionalizes equity as a measurable component of health system quality.

## From Ethical Mandate to a Health Standard

Addressing algorithmic bias should not be seen as a barrier to innovation. On the contrary, addressing such bias should be considered a catalyst for better science. An algorithm rigorously tested and validated to perform equally across diverse populations is inherently more robust, reliable, and effective. Confronting and correcting these biases allows AI to serve a dual purpose: improving health outcomes and exposing the hidden disparities within health systems, using technology as a mirror to reveal where actions, applications, and policies must improve. Embedding bias detection into national quality standards and linking fairness to funding eligibility can institutionalize fairness as a measurable component of health system performance.

## Conclusion and Call to Action

As the region of the Americas advances its digital transformation, the Pan American Health Organization urges countries, developers, and regulators to treat algorithmic fairness not as a technical afterthought but as a foundational element of resilient and inclusive health systems. This imperative aligns directly with the Pan American Health Organization’s *Eight Guiding Principles of the Digital Transformation of the Health Sector,* notably Principle 2: Equity and Inclusion, which emphasizes the need to ensure that digital health benefits reach all population groups, and Principle 5: Ethical Use of Technology, which calls for the responsible and transparent design of digital tools [21]. Addressing algorithmic bias, from data collection to real-world deployment, is an ethical and health mandate that underpins scientific reliability, system trustworthiness, and equitable health outcomes.

Ensuring fairness also means that AI strengthens primary health care and essential public health functions, not only specialized clinical services, and future work should continue to explore how AI can be developed and deployed in ways that reinforce these levels of care, where evidence generation and data availability have historically been more limited.

AI holds extraordinary potential to enhance early detection, optimize resources, and extend care to underserved populations. But if left unchecked, it risks reinforcing the same data-driven health disparities that public health aims to dismantle. Ensuring that AI systems are designed to recognize and counter bias from the start will help create health technologies that are not only more accurate and reliable, but also truly reflective of the populations they aim to serve.

## Abbreviations

AI: artificial intelligence
